# Transition to organic farming negatively affects bat activity

**DOI:** 10.1111/1365-2664.14468

**Published:** 2023-07-19

**Authors:** Penelope C. Fialas, Jérémy S. P. Froidevaux, Gareth Jones, Péter Batáry

**Affiliations:** ^1^ School of Biological Sciences University of Bristol, Life Sciences Building Bristol UK; ^2^ College of Life and Environmental Sciences University of Exeter, Hatherly Building Exeter UK; ^3^ Centre d'Ecologie et des Sciences de la Conservation (CESCO, UMR 7204), CNRS, MNHN, Sorbonne‐Université Concarneau France; ^4^ Biological and Environmental Sciences University of Stirling Stirling UK; ^5^ ”Lendület” Landscape and Conservation Ecology Institute of Ecology and Botany, Centre for Ecological Research Vácrátót Hungary

**Keywords:** agri‐environment schemes, bat activity, farming system, landscape complexity, time‐lag, transition period

## Abstract

1. The effectiveness of organic farming on biodiversity has been widely documented especially for plants, arthropods and birds; however, the effects of the transition period required to become an organic farm on wildlife remain poorly understood.
2. We assessed the effects of organic farming on insectivorous bats in citrus orchards in the Republic of Cyprus employing two matched designs (conventional vs. 3‐year organic‐transitional and conventional vs. organic‐certified) and a third unmatched design (3‐year organic‐transitional vs. organic‐certified). We specifically investigated whether the transition period prior to full organic certification influenced bat activity with a special focus on any moderation effects from surrounding semi‐natural areas.
3. The activity of three (*Pipistrellus kuhlii, Hypsugo savii* and *Miniopterus schreibersii*) of four bat species was significantly lower in farms undergoing the transitional period than in conventional farms, and *P. kuhlii and H. savii* were significantly less active in organic transitional farming systems that in organic‐certified ones. Furthermore, the activity of the most dominant species (*P. kuhlii*) was significantly higher on organic than transitional and conventional citrus orchards, thus suggesting a time‐lag effect. Landscape complexity measured as the amount of semi‐natural areas did not moderate the effects of farming system for any study species.
4. *Synthesis and application*. The transition to organic farming had persistent detrimental effects on bats and potentially on the pest suppression services they provide. Future agri‐environmental policy should consider the transition period and implement measures to mitigate any negative effects on biodiversity, alongside promoting asynchronous transition of nearby farms. Our findings further highlight the crucial need to consider the time since transition to organic farming when assessing potential benefits of organic management on biodiversity.

## Graphical abstract

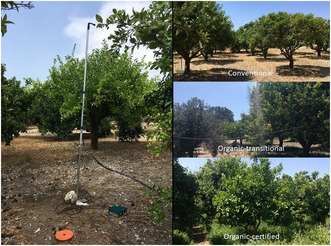

## 1 INTRODUCTION

Agriculture is a dominant land use worldwide and is the primary land use type in the enlarged European Union (EU27) covering 45% of land cover (180 million ha; Food and Agriculture Organization of the United Nations, 2005), and mostly involving intensive agricultural practice (Stoate et al., 2001). A direct consequence of the intensification and expansion of modern agricultural practices in the last century is the biological simplification of the farmed environment, becoming increasingly visible through declines in farmland biodiversity and reduced compositional and configurational landscape heterogeneity (Fahrig et al., 2011; Fuentes‐Montemayor et al., 2011; Reif & Vermouzek, 2019; Traba & Morales, 2019; Tscharntke et al., 2005). In addition, there are severe losses in regulatory ecosystem services, including biological pest control (Bianchi et al., 2006; Weibull et al., 2003).

Agri‐environment schemes (AESs) have been developed and introduced by the European Union to counteract the negative environmental effects of conventional farming on biodiversity (Batáry et al., 2015), and organic farming is one of the most well‐established AES management approaches of the member states (Batáry et al., 2013). Organic farming is a production system that sustains the health of soils, ecosystems, and people relying on ecological processes, biodiversity and cycles adapted to local conditions, rather than the use of inputs with adverse effects (Willer et al., 2021). As a result, the certified organic and in conversion area within the EU increased from 10.04 million hectares in 2012 to 13.43 million hectares in 2018 representing 7.5% of the utilised agricultural area in Europe (EU28; EUROSTAT, 2018).

There is a significant and still growing body of studies investigating the effects of organic management on biodiversity (Barbaro et al., 2021; Katayama et al., 2019; Rollan et al., 2019; Rundlöf & Smith, 2006; Schneider et al., 2014; Wintermantel et al., 2019), and it is now widely accepted that (i) the effects of organic farming on biodiversity strongly vary among taxa (Fuller et al., 2005; Winqvist et al., 2012); (ii) the direction and magnitude of these effects can be affected by the character of the surrounding landscape and the spatial scale considered (Batáry et al., 2010; Rundlöf & Smith, 2006; Tscharntke et al., 2012a; Tuck et al., 2014) and (iii) landscape diversification could potentially be more important than conversion of conventional to organic farming (Tscharntke et al., 2021). The potential time‐lag in the response of biodiversity to organic farming could also be an important driver of variation in organic management outcomes (Watts et al., 2020), even though there is so far little evidence (Andersson et al., 2010; Jonason et al., 2011). In fact, depending on the species group examined (e.g. less mobile vs. more mobile species) and the measure implemented (e.g. habitat restoration vs. changes in habitat management), the benefits of organic farming may vary over time. Besides, research on the effects of organic farming on biodiversity in the Mediterranean bioclimatic region has been particularly limited despite it being a biodiversity hotspot (Myers et al., 2000). This is especially true for the Mediterranean islands, which host the highest richness of threatened or endemic species for all taxa in Europe (Maiorano et al., 2013).

To become organic certified, a transition period is required. It lasts a minimum of 3 years and involves specific constraints and procedures. Those mainly involve avoidance of any synthetic inputs at all stages (e.g. chemical fertilisers and pesticides), taking precautionary measures to avoid contamination; assuring long‐term biologically based soil fertility and employ long‐term, ecological, system‐based organic management (e.g. increase plant‐based ground cover and enhance biodiversity; see details in Table S1 in Supporting Information). During this transition period, severe weed infestation, inadequate soil fertility and other pest problems often occur, resulting in economic losses (Bellon & Lamine, 2009; Dabbert & Madden, 1986). However, the sole focus of these studies was to assess the impact of organic farming during the transition periods on yields for economic purposes. Although one would expect that in the absence of use of agrochemicals the transitional farms have neutral to positive effects on vertebrates, this may not be the case as negative effects at the bottom of the food chain could potentially negatively affect higher trophic levels (Tsutsui et al., 2018). Invertebrate abundance declines during the transition phase that organic farming requires before farms are fully certified after a changeover from conventional farming (Andersen & Eltun, 2000), and this transition can cause a non‐equilibrium that could last for a decade (Tsutsui et al., 2018). However, none of these studies has examined the effects on vertebrates. Therefore, an understanding of the effects on biodiversity during this transition period is of crucial importance to provide information about the suitable conversion strategies necessary to implement and minimise any potential negative effects.

Insectivorous bats are an important component of agricultural landscapes and play an important role as bioindicators (Jones et al., 2009; Park, 2015) and in pest suppression (Aizpurua et al., 2018; Charbonnier et al., 2021; Cohen et al., 2020; Kolkert et al., 2020; Maslo et al., 2022; Puig‐Montserrat et al., 2020; Russo et al., 2018). Studies assessing the effects of a range of agricultural management practices on bats have reported inconsistent results. Some studies highlighted significant positive impacts of organic farming on some bat species (Barré et al., 2018; Lesiski et al., 2013; Puig‐Montserrat et al., 2021; Rodríguez‐San Pedro et al., 2018; Toffoli & Rughetti, 2017; Wickramasinghe et al., 2003), whereas other studies found no significant effect (Davy et al., 2007; Froidevaux, Louboutin, & Jones, 2017; Long & Kurta, 2014; MacDonald et al., 2012; Pocock & Jennings, 2008).

In this study, we use bats as model taxa to investigate the effects of organic farming with its transitional phase on biodiversity. Our main objective was to evaluate the effect of three different farming systems (conventional, organic‐transitional, organic‐certified) on bat activity in citrus orchards in Cyprus. We predicted that bat activity would be higher on organic farms (both transitional and certified) compared to conventional farms due to the use of pesticides in conventional farms, resulting in a decrease in arthropods having cascading effects on bats (Wickramasinghe et al., 2004). Our second objective was to assess the interplay between agricultural management and landscape simplification in driving bat activity in citrus orchards. We predicted that the benefits of organic farming would be higher in landscapes of intermediate complexity based on the ‘intermediate landscape complexity’ hypothesis (Tscharntke et al., 2012b) that states that landscape‐moderated effectiveness of local conservation management (i.e. organic farming) is highest in structurally simple, rather than extremely simplified or complex landscapes.

## 2 MATERIALS AND METHODS

### 2.1 Study area and sampling design

We conducted the study in the Republic of Cyprus (Figure S1 in Supporting Information) (licence: 2.17.3/15, Ministry of Agriculture, Rural Development and Environment), where organic farming as part of national AES had increased rapidly (over 38×) during the last two decades, comprising 4.6% of the agricultural land in 2020 compared to 0.12% of agricultural land in 2002 when it was first initiated (Ministry of Agriculture, Rural Development and Environment, 2020 unpublished). Sampling took place in 2017 during two critical periods for bats: (i) lactation period: mid‐July to early August and (ii) post‐lactation period: mid‐September to early October. We surveyed a total of 22 matched‐pairs of citrus orchards, comprising 11 certified organic farms matched with 11 conventionally managed control farms (period 1: 7 farms; period 2: 4 farms), and 11 organic‐transition farms matched with 11 conventionally managed control farms (period 1: 4 farms; period 2: 7 farms). Organic farmers were certified by the organic certifying bodies in Cyprus (Lacon Ltd and Biocert Ltd) following the criteria of the Cypriot Council Regulation (EC) No 834/2007 (see Table S2 for detailed information regarding the criteria). To avoid any confounding variables affecting any real differences due to farm management, we selected citrus orchards (mean area: 0.77 ha; range: 0.26–4.06 ha) with similar landscape and local habitat characteristics in a pair (see Section 2.3 and Table S3 in Supporting Information). Sampling sites were located at the centre of each orchard plot and were situated at least 400 m away from each other (mean: 1495 m; range: 428–7000 m). No ethical approval was required. Access to sites was granted by private property landholders.

### 2.2 Bat echolocation call recording and identification

We acoustically sampled bats using two Song Meter SM2BAT+ bat detectors (sampling rate: 384 kHz; Wildlife Acoustics) connected to SMX‐US ultrasonic microphones mounted on 3 m poles, facing upwards at 45°. We programmed the detectors to automatically record sounds in the frequency range 12–192 kHz and ≥12 dB above background noise. Each recording sample lasted for 15 s. Plots within a pair were simultaneously surveyed during one full night, from 30 min before sunset until 30 min after sunrise. We switched detectors between each survey night to avoid any bias caused by possible differences in microphone sensitivity. Sampling was conducted only during good weather conditions (minimum temperature at night >15°C; wind speed ≤4 on Beaufort scale). We measured temperature each night every 15 min using a Data Logger RC‐5 (accuracy: ±0.5°C; Elitech), mounted at the 3 m poles. In case of missing data (<30%), we used data provided by the Department of Meteorology, Cyprus.

We manually identified each bat pass—defined as a series of minimum two echolocation calls with pulse interval (s) <1—using a full spectrum, 15 s WAV file in BatSound 4.1.4. (Pettersson Electronic). Over 90% of bat passes were identified to species level while ambiguous calls were assigned to species‐complex (e.g. *Pipistrellus pipistrellus*/*P. kuhlii*) or grouped by genus (e.g. *Myoti*s spp). Identification criteria were based on call characteristics (see Appendix S2 for details). We used bat activity (i.e. number of bat passes) as a surrogate of bat abundance (Froidevaux, Boughey, et al., 2017).

### 2.3 Local habitat structure and landscape analysis

To control for any potential cofounding variables known to drive bat activity, we conducted local and landscape analyses.

We delimited a stand of 15 × 15 m around each sampling site (i.e. centre of the citrus orchard where bat detector was installed) to document the local habitat variables, including tree height and ground vegetation cover (Table S5 in Supporting Information). Local habitat variables collected at the stand scale were representative of the farm. We also noted the presence/absence of linear features (i.e. hedgerow or tree line) along field boundaries. There was little variation in tree height and this variable was disregarded for the statistical analysis. To get finer details on ground vegetation cover, data were collected within each of the nested square (7.5 × 7.5 m). Only one surveyor (PCF) conducted the survey, thus eliminating potential observer bias.

We extracted landscape variables within three buffers (1, 2 and 3 km radii) that we created around the sampling sites using ArcGIS Desktop 10.5 (ESRI; Figure S2 in Supporting Information). The scales were selected to represent both site‐specific characteristics (1 km) and main core foraging zones of bats (2 and 3 km; Laforge et al., 2021). We reclassified feature classes obtained from CORINE Land Cover data 2012 supplied by the European Environment Agency (www.eea.europa.eu) into 10 categories: urban area, arable, vineyard, fruit orchard, olive, other agricultural area, mixed and deciduous forest, coniferous forest, semi‐natural area and water bodies (Table S6 in Supporting Information). Then we calculated the amount of semi‐natural habitats (natural grasslands, moors and heathland, sclerophyllous vegetation and transitional woodland shrub) and urban areas at each spatial scale. While semi‐natural habitats represent important foraging habitats in Mediterranean landscapes for many bat species, urban areas can be used as a proxy of roost availability for the most common species occurring in the study area such as *P. kuhlii* and *P. pipistrellus* that are known to roost in man‐made structures (Dietz et al., 2009). To take into account the extensive use of water bodies by bats especially in areas with a Mediterranean climate (Cruz et al., 2016; Russo & Jones, 2002; Sirami et al., 2013), we calculated for each sampling site the Euclidean distance to the nearest main river crossing the study area (i.e. Pedieos and Yalias) and the nearest dams and reservoirs.

### 2.4 Statistical analysis

We performed two separate set of analyses to assess the effects of farming system (conventional vs. organic‐transitional, conventional vs. organic‐certified and organic‐transitional vs organic‐certified) on bat activity. In the first set of analyses, we used a series of generalised linear mixed‐effect models (GLMMs; lme4 package; Bates et al., 2015) and included the farm system pair (conventional vs. organic‐certified and conventional vs. organic‐transitional) in interaction with the amount of semi‐natural habitats within 2 km radius buffer scale (i.e. proxy of landscape complexity) as independent variables (see Figure S3 in Supporting Information) and pair ID as a random effect. In the second set of analyses, we ran generalised linear models with the farming system of organic‐transitional vs. organic‐certified as interactions with the amounts of semi‐natural habitats as independent variables. We also included several covariates to control any confounding factors as this pair was not controlled in our design, namely structural orchard features (presence/absence of woody linear feature, ground vegetation cover), landscape characteristics (amount of semi‐natural habitats, urban areas and distance to the nearest water bodies) and Julian days. We compared a series of models containing all possible preselected predictor variable combinations (see Appendix S2 for more details) using the *dredge* function (mumin package; Bartoń, 2016) for model selection. From the most parsimonious models, we only retained the models with the lowest number of parameters and (whenever possible) that included farming systems (i.e. the variable of interest; Table S9 in Appendix S1). We considered activity of the most active species (i.e. *Pipistrellus pipistrellus, P. kuhlii, Hypsugo savii* and *Miniopterus schreibersii*) as response variables in our models. Models were fitted with the appropriate error distribution, that is Poisson or negative binomial family, when overdispersion was present, with a logit link function (Zuur et al., 2009).

Models were diagnosed and validated using the dharma package (Hartig, 2018). Residual spatial autocorrelation in the final GLMMs models was inspected using Mantel tests and no spatial autocorrelation was found (Table S7 in Supporting Information). We used output from the full models and considered an effect to be significant at *p* < 0.05.

We have checked the consistency of our results by implementing an alternative approach using the whole dataset in a unique model framework (see Appendix S2 form more details). This led to similar results (Table S1 in Appendix S2). All statistical analyses were conducted in R version 4.2.0 (R Development Core Team, 2022).

## 3 RESULTS

### 3.1 Bat acoustic sampling

We recorded 9826 bat passes from nine taxa (Table S4) within 44 citrus orchard plots (22 conventional, 11 certified‐organic and 11 transitional‐organic). Nearly 88% of all bat passes (8461 passes) belonged to the *Pipistrellus* genus, with *P. kuhlii* being 70% more frequently recorded than *P. pipistrellus*. Only four out of nine species detected were retained for analysis due to the fact of insufficient data for the remaining species.

### 3.2 Effects of farming system on bats

**FIGURE 1:**
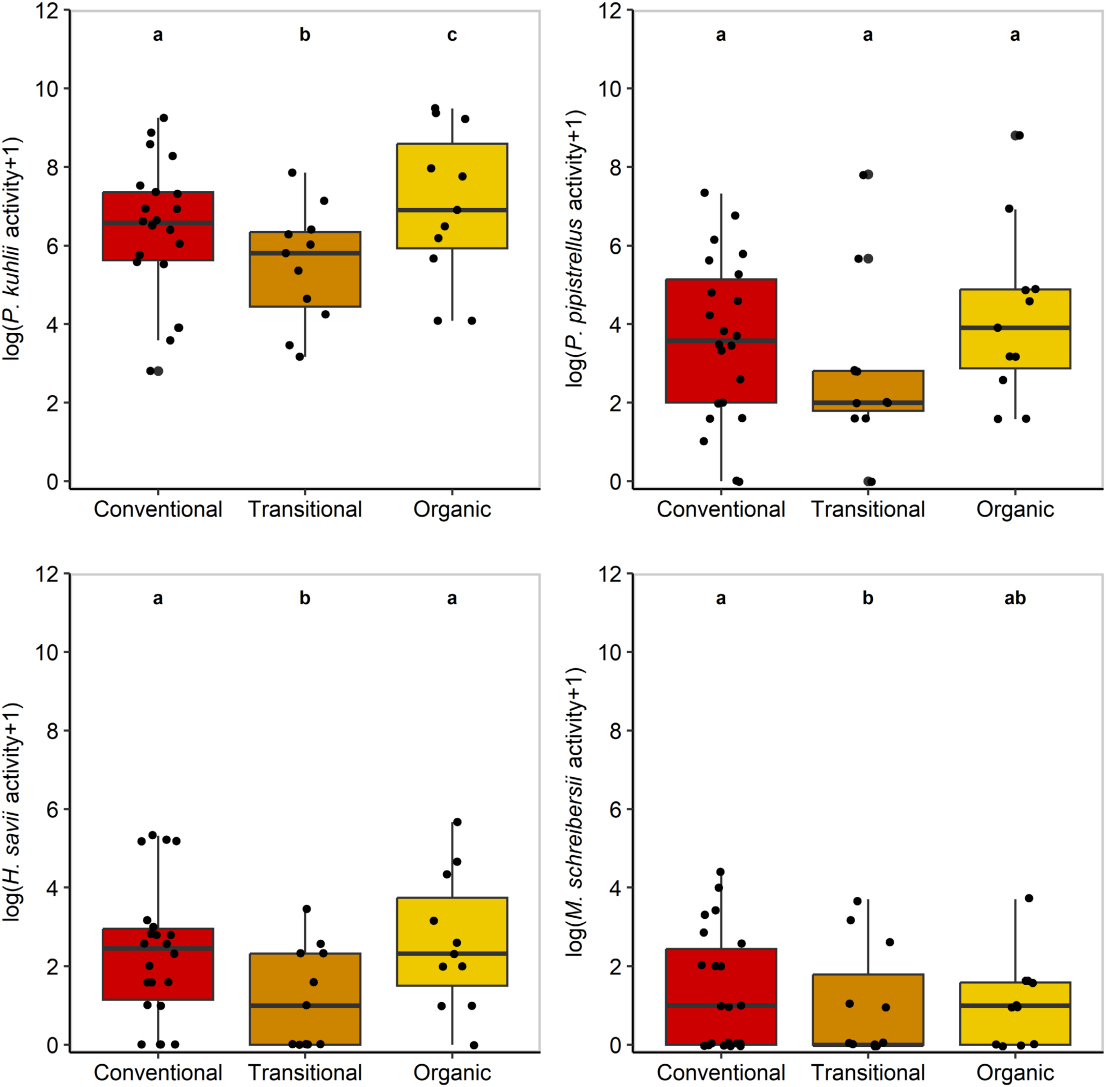
Boxplots showing medians and interquartile ranges of the activity (i.e. total number of bat passes per site on a logarithmic scale to the base 2) of four bat species (*Pipistrellus kuhlii*, *Pipistrellus pipistrellus*, *Hypsugo savii* and *Miniopterus schreibersii*). Black dots represent the raw data. Superscripts a and b are used to identify statistically significance differences between farming systems. Statistical significance arises from three different models (see Section 2.4).

For the first set of analysis, our results showed that *P. kuhlii, H. savii* and *M. schreibersii* were significantly less active in organic‐transitional citrus orchards in comparison with conventional ones (Figure 1; Table 1). Our models suggested that *P. kuhlii* and *M. schreibersii* activity was more than twofold lower in organic‐transitional compared to conventional farming system and *H. savii* threefold. Furthermore, we observed a significant positive effect of organic‐certified farming compared to conventional farming system only for *P. kuhlii* activity, showing that *P. kuhlii* was twofold higher in organic‐certified citrus orchards than conventional ones. For the four species, models with only farming system were more informative than the model with the landscape complexity and the null model with dAICc <2 (Table S8).

**TABLE 1:** Summary results from generalised linear mixed models (Model 1 and Model 2) and generalised linear models (Model 3) of the two set of analysis (see Section 2.4) testing the effects of farming system on bat activity. Estimates, associate standard error (SE), 95% confidence intervals, marginal (variance explained by the fixed effects only) and conditional (variance explained by both fixed and random effects) *R* ^2^ values are given. Explanatory variables displayed in bold represent significant variables for which 95% CI did not overlap zero.

Response variable	Model	Explanatory variable	Estimate	SE	Lower 95 CI	Upper 95 CI	*p*	m*R* ^2^	*cR* ^2^
*Pipistrellus kuhlii*	Model 1	**Organic (C) vs. Conventional**	0.65	0.18	0.30	1.00	***	0.06	0.90
	Model 2	**Organic (T) vs. Conventional**	−0.89	0.39	−1.64	−0.13	*	0.14	0.20
	Model 3	**Organic (T) vs. Organic (C)**	−1.32	0.42	−2.15	−0.49	**	0.41	
*Pipistrellus pipistrellus*	Model 1	Organic (C) vs. Conventional	0.18	0.44	−0.68	1.04	NA	0.04	0.44
	Model 2	Organic (T) vs. Conventional	−0.75	0.68	−2.07	0.57	NA	0.03	0.22
	Model 3	Organic (T) vs. Organic (C)	−0.70	0.53	−1.73	−0.33	NA	0.52	
*Hypsugo savii*	Model 1	Organic (C) vs. Conventional	0.15	0.13	−0.11	0.41	NA	0.27	0.95
	Model 2	**Organic (T) vs. Conventional**	−1.32	0.64	−2.56	−0.07	*	0.08	0.08
	Model 3	**Organic (T) vs. Organic (C)**	−1.51	0.59	−2.66	−0.36	*	0.21	
*Miniopterus schreibersii*	Model 1	Organic (C) vs. Conventional	0.09	0.47	−0.83	1.01	NA	0.23	0.42
	Model 2	**Organic (T) vs. Conventional**	−1.19	0.44	−2.05	−0.33	***	0.36	0.92
	Model 3	Organic (T) vs. Organic (C)	/	/	/	/		/	/

Our results when comparing organic‐transitional to organic‐certified (second set of analysis) showed that *P. kuhlii* and *H. Savii* were significantly lower in organic‐transitional orchards compared to organic‐certified ones by threefold and twofold, respectively (Figure 1; Table 1). Farming system was retained in almost all most parsimonious models, except for models on *M. schreibersii* activity (see Table S9 in Supporting Information).

### 3.3 Testing the intermediate landscape hypothesis

The interaction between farming system and landscape complexity (i.e. % of semi‐natural habitat) was never significant. Furthermore, landscape complexity on its own had a negative effect on *P. kuhlii* and a nearly significant effect on *P. pipistrellus* and *H. savii* when comparing organic certified farming systems with conventional ones. However, the models including landscape complexity were less informative than the models with only farming system (see Table S8 in Supporting Information).

## 4 DISCUSSION

The effects of the transition phase required prior to becoming a certified organic farm have, to the best of our knowledge, never been analysed before on vertebrates. In this study, we provide empirical evidence of the effects of farming systems (conventional, organic‐transitional, organic‐certified) considering the transition phase on bat activity in Mediterranean citrus orchards. We unexpectedly found that bat activity in the organic‐transitional farming system was significantly lower than in certified organic orchards and conventionally farmed orchards. Furthermore, our findings on the most active species highlight the importance of time‐lags in how species respond to organic management.

### 4.1 Effects of farming system on bats

Our results indicated that *P. kuhlii, H*. savii and *M. schreibersii* activity in Mediterranean citrus orchards was PERSISTENGLY negatively affected during the transition to organic farming. This could have high impact to bat populations as they lose important foraging grounds. For example, Andersen and Eltun (2000) showed that in organic‐transitional farming there was a significant decrease in the abundance of some insect species, which could be possible prey of bats. This can be ultimately linked to soil fertility determining also insect abundance several studies have emphasised that soil nutrient deficiency and inadequate soil fertility occur during this transition period (Bellon & Lamine, 2009; Borrelli et al., 2012; Dabbert & Madden, 1986; Dalgaard et al., 2002; Gopinath et al., 2008). Soil microbial biomass is important to supply plant nutrients by mineralisation processes and to avoid nutrient leaching (Friedel et al., 2001). In addition, soil macronutrient deficiency can have negative effects on insect abundance, species richness and composition (De Araújo, 2017; Haddad et al., 2000; Tsutsui et al., 2018) and subsequently on the foraging behaviour of insectivorous species, such as bats, creating a bottom‐up cascading effect (Basham et al., 2011; Threlfall et al., 2011, 2012). Furthermore, Law and Chidel (2001) found a positive association between nutrient rich soil and high bat activity. These transition years pose many challenges, because the changes in the chemical, physical and biological properties of the soil take time to reach an ecological balance (Gopinath et al., 2008).

An alternative explanation for the negative effect of transition farming could be due to an ‘organic transition effect’ proposed by Dabbert and Madden (1986). During the transition period, ecological processes are inadequate to supply nutrients to control pests and diseases, or to provide essential functions previously provided by chemical inputs. This effect would be stronger in farms that previously used high‐intensity agricultural practices (Bellon & Lamine, 2009; Borrelli et al., 2015). Some studies suggest that soil quality and biological activity improve only after three or more years of organic management (Bellon & Lamine, 2009; Dabbert & Madden, 1986; Riedo et al., 2022; Tsutsui et al., 2018; Tu et al., 2006).

The effects of organic farming on bats are still not well understood, with studies showing contrasting findings. In this study, we found that organic citrus orchards compared to transitional and conventional citrus orchards enhance *P. kuhlii* activity and this species dominated the species assemblage. These results are in accordance with previous studies (Barré et al., 2018; Fuller et al., 2005; Put et al., 2018; Wickramasinghe et al., 2003), which highlight the positive influence of organic farming on bats. However, there were no statistically significant effects on the other bat species, which corroborate with studies that have found no effect of organic farming on bats (e.g. Froidevaux, Boughey, et al., 2017). Organic farming effects on biodiversity might be specific to taxonomic and functional groups (Tuck et al., 2014). Moreover, these variable results could be attributed to the ‘colonisation credit’ (With, 2007), where there is a time‐lag in species response following landscape changes (e.g. land use changes caused by AESs). That is, even if a patch increases its quality (i.e. from conventional to organic farming), it will take some time before organisms colonise the patch (Andersson et al., 2010). The length of the time‐lag will depend on multiple factors such as species dispersal ability, mechanisms operating behind species responses at the individual and population level, on the patch's spatial properties (e.g. landscape connectivity) and on the magnitude of the changes (e.g. habitat restoration vs. changes in habitat management; Cristofoli et al., 2010; Jonason et al., 2011; Watts et al., 2020). In addition, pesticide residues in soil could explain why there is a delayed/gradual response of some organisms (Silva et al., 2019). Transitional farming could potentially still contain pesticide residues, especially in intensively managed permanent crops, persisting for over 20 years, affecting the response of some organisms such as arthropods (Riedo et al., 2022) and potentially affecting negatively bat activity as a consequence. Tsutsui et al. (2018) found that a non‐equilibrium state of arthropod populations due to residual pesticides could last for a decade after the transition to organic farming.

### 4.2 Intermediate landscape complexity hypothesis

Our results did not find any significant effect regarding the interaction of landscape complexity and farming systems, thus not supporting the ‘intermediate landscape complexity’ hypothesis that states that local conservation management (e.g. organic farming) within the agricultural matrix is most effective in landscape of intermediate complexity (1%–20% non‐crop habitat) compared to cleared landscapes (extremely simplified; <1% non‐crop habitat) or in complex landscapes (>20% non‐crop habitat). However, this only assessed the linear effect, without considering the form of such interaction. Nevertheless, these results corroborate those of Froidevaux, Boughey, et al. (2017) who found no significant relationship between landscape complexity and farming system. In contrast, Boonchuay and Bumrungsri (2022) found that bat activity was significantly higher in organic rice paddies in simple landscape indicating that farming system depends on the level of landscape complexity, thus supporting the ‘intermediate landscape complexity’ hypothesis. These contrasting results would suggest that any effect of landscape complexity is dependent on crop type and context.

### 4.3 Implications for conservation and management

The transition phase is an essential step required for all organic farming systems; however, its effects on vertebrates have not been assessed previously. Our results suggest that the transition to organic farming may have potential persistent detrimental effects on bat activity and this could be the case for broader biodiversity and could be also applied to other slow developing organic crops such as olive groves and vineyards. Olive groves and vineyards represent one of the main crop systems in the Mediterranean basin. Olive orchards cover approximately 10 million hectares worldwide and 90% are located in the Mediterranean basin and vineyards cover over 3 million hectares in Europe (Food and Agriculture Organization [FAO], 2018).

Simple comparisons of organic and conventional farming are not enough especially in the light of possible strong increase in the share of organic farming according to the Green Deal of the EU. We therefore urge future studies to investigate the effects of transition organic farming on other taxa with different functional traits (mobility, dispersal ability and home range size). Given that organic farming is one of the most popular AESs and is growing continuously, the findings of this study could have high relevance to farmers and agricultural policymakers. We recommend that future AESs should promote asynchronous transition of nearby farms, especially in countries (e.g. Cyprus) where the number of organic farms is rising drastically, to avoid having large areas within a landscape under transition. We recommend that future AESs should consider time‐lags of population responses to land‐use changes to capture full potential of such schemes as it might take some time before species can respond and for the potential benefits to be manifested. Hence, these schemes should be sustained for a long period of time. To evaluate the costs and benefits of organic farming, it is necessary to consider the time‐dependent responses of organisms. Using such an evaluation could help establish guidelines pertaining to how long organic farming should continue to restore biodiversity or whether an economic subsidy should be provided to compensate for pests, weed infestations and other factors.

Moreover, AESs should provide suitable conversion strategies to mitigate the potential negative effects it could have on biodiversity during this critical period of transition by adopting a multi‐scale approach, which could provide benefits to bats for example by (i) increasing connectivity to the source habitats of some arthropods as this could restore these arthropod populations, especially in complex landscapes with larger species pools; (ii) forming water bodies in the vicinity of citrus orchards (Lisón & Calvo, 2011; Russo & Jones, 2003) and (iii) increasing the abundance and diversity of herbaceous vegetation as this will increase arthropod diversity and enhance soil conditions (Silva et al., 2010), which is an important factor during the transition period. These strategies will not only mitigate the short‐term detrimental effects of transitional organic farming, but will also increase bat activity and broader biodiversity in the long run.

## AUTHOR CONTRIBUTIONS

Penelope C. Fialas and Peter Batáry conceived the idea and along with Jeremy S. P. Froidevaux co‐designed the study; Penelope C. Fialas collected and along Jeremy S. P. Froidevaux analysed the field data and Penelope C. Fialas led the writing of the manuscript. Gareth Jones provided supervision, equipment and laboratory facilities. All authors contributed critically to the drafts and gave final approval for publication.

## CONFLICT OF INTEREST STATEMENT

The authors have no conflict of interest. Peter Batáry is an Associate Editor of *Journal of Applied Ecology* but took no part in the peer review and decision‐making processes of this paper.

## Supporting information


**Appendix S1.** Supplementary material.

**Appendix S2.** Alternative statistical approach.

## Data Availability

Data available via the Dryad Digital Repository: https://doi.org/10.5061/dryad.rjdfn2zhx (Fialas et al., 2023).
