# Supplementary material for: Transition to organic farming negatively affects bat activity
Source: J Appl Ecol. 2023 Jul 19;60(10):2167–76. doi: 10.1111/1365-2664.14468 (PMC10947233; doi:10.1111/1365-2664.14468)
Supplement: Supplementary file 1 — Appendix S1. Supplementary material. [file JPE-60-2167-s002.pdf]

Appendix_S1_Supplementary material

Transition to organic farming negatively impacts bats and leads to a time-lag in their responses to farm management

Penelope C. Fialas[1,2*], Jérémy S. P. Froidevaux[1,3], Gareth Jones[1,c] & Péter Batáry[4,c]

[*]Corresponding author: University of Bristol, School of Biological Sciences, Life Sciences Building, 24 Tyndall Avenue, BS8 1TQ Bristol, United Kingdom.

Tel: +44(0)11.73.94.13.86. E-mail: pf16461@bristol.ac.uk

Table S1. Transition/Conversion Requirements (Council Regulation (EK) No. 834/2007)

**Transition/Conversion Requirements**
Organic guarantee systems clearly identify when organic practices begin and how long they are applied before the operation and products can be considered organic. This may include specific conditions for simultaneous transition/conversion of land and animals.
For crops, organic guarantee systems establish a suitable period of time prior to the organic status of a crop, (during which healthy soils and sustainable ecosystems are being established).<br>• Common minimum time periods:<br>a) organic management for least 12 months for annuals and 18 months for perennials.<br>b) 36 months since application of any inputs that do not accord with organic principles and applicable standards.
Organic guarantee systems require that animal production systems raise animals organically from birth or hatching, or when this is not possible from early ages subject to a minimum transition/conversion requirement.<br>• Common minimum transition/conversion requirements: dairy – 90 days; eggs and poultry meat – 42 days; other meat – 12 months; bee colonies – time needed for wax replacement with minimum twelve months
The conversion period shall start at the earliest when the operator has notified his activity to the competent authorities and subjected his holding to the control system in accordance with Article 28(1)
in order to determine the conversion period referred to above, a period immediately preceding the date of the start of the conversion period, may be taken into account, in so far as certain conditions concur
During the transition/conversion period all regulations established by Council Regulation (EK) No. 834/2007 shall apply
On a holding or unit partly under organic production and partly in conversion to organic production, the operator shall keep the organically produced and in-conversion products separate and the animals separate or readily separable and keep adequate records to show the separation;
The measures and conditions necessary for the implementation of the rules contained in this Article, and in particular the periods referred to in paragraph 1(c) to (f) shall be defined in accordance with the procedure referred to in Article 37(2).

Table S2 Regulations and requirements to become Organic farmer (Council Regulation (EK) No. 834/2007)

Main Objectives	Detailed requirements
**1. Employ long-term, ecological, systems-based organic management.**	Organic management does not rely upon switching back and forth between organic and conventional management.
	Organic crop production systems conserve or improve the soil's structure, organic matter, fertility and biodiversity.
	Organic crop production management includes a diverse planting scheme as an integral part of the system of the holding. For perennial crops, this includes plant-based ground cover
	Organic crop production management employs interrelated positive processes and mechanisms for the management of pests, diseases, and weeds.
**2. Assure long-term, biologically based soil fertility.**	Organic crop production systems enhance soil primarily by incorporating manures and other biodegradable inputs, and/ or by nitrogen fixation from plants.
	Organic soil fertility management uses only naturally occurring mineral fertilizers and only as a supplement to biologically-based fertility methods. Organic crop production does not use sodium (chilean) nitrate.
**3. Avoid/minimize synthetic inputs at all stages of the organic product chain and exposure of people and the environment to persistent, potentially harmful chemicals.**	Organic soil fertility management does not use synthetic fertilizers or fertilizers made soluble by chemical methods, e.g. superphosphates and uses only crop fertility substances that are on (a) list(s) maintained by the standard.
	For food and feed production, organic processing uses only processing methods that are biological and physical in nature.
	Organic management takes precautionary measures to avoid contamination (commonly this includes barriers/buffers in production, cleaning of farm equipment, separation and cleaning in processing).

	Organic processing management identifies and minimizes risks of product contamination.
**4. Minimize pollution and degradation of the production/processing unit and surrounding environment from production/processing activities.**	Organic management maintains or enhances biodiversity on the farm holding, in crop and non-crop habitats.
	Organic crop production systems employ measures to prevent land degradation, such as erosion and salinization.
	Organic management ensures that water resources are used sustainably.
	Organic management does not undertake any actions that negatively impact high conservation value areas.(E.g Natura 2000 areas)
	Organic guarantee systems restrict use of synthetic coverings and mulches in organic production systems.
**5. Exclude certain unproven, unnatural and harmful technologies from the system.**	All organic management systems do not use genetically modified organisms (GMO) or their derivatives, except vaccines, in all stages of organic production and processing.
	Organic processing does not use irradiation (ionizing radiation) technologies.

http://www.moa.gov.cy/moa/da/da.nsf/page22_gr/page22_gr?OpenDocument

Table S3. Pairwise comparison of plot and landscape variables assessed during fieldwork preparation to obtain adequate pairs of organic and conventional citrus orchards. None of the comparisons are statistically significant (*P*-values > 0.05).

Scale	Variable	Conventional citrus orchard mean (± SE)	Organic (C) citrus orchards mean (± SE)	Organic (T) citrus orchards mean (± SE)	Test	P Conv-Org (C)	P Conv-Org (T)
Plot	Area (ha)	0.75 ± 0.13	0.7 ± 0.1	0.9±0.32	Paired t-test	0.8639	0.6185
Plot	Altitude (m a.s.l.)	125.45 ± 20.74	186.36 ± 38.45	137.82 ± 35.93	Paired t-test[b]	0.6536	0.1752
Landscape	% of forest and semi-natural habitats within 1.0 km radius buffer	10.15 ± 3.94	16.53 ± 6.76	6.85 ± 3.46	Permutation test/ Paired t-test[b]	0.4006	0.117
Landscape	% of forest and semi-natural habitats within 2.0 km radius buffer	18.86 ± 6.38	19.88 ± 6.94	12.86 ± 4.11	Paired t-test[b]	0.5298	0.4875
Landscape	% of forest and semi-natural habitats within 3.0 km radius buffer	20.46 ± 4.63	24.42 ± 6.86	18.43 ± 4.8	Paired t-test	0.5648	0.822
Landscape	Distance to urban areas (km)	1.15 ± 0.21	1.04 ± 0.29	0.74 ± 0.16	Paired t-test	0.1609	0.2708
Landscape	Distance to dams (m)	0.06 ± 0.01	0.07 ± 0.01	0.04 ± 0.01	Permutation test/Paired t-test	0.976	0.3046
Landscape	Distance to main rivers (m)	0.21 ± 0.05	0.09 ± 0.03	0.33 ± 0.08	Paired t-test/Permutation test	0.8048	0.9713

[a] Paired t-test when data were normally distributed; Permutation test otherwise (Monte Carlo permutation test for paired individual scores; “surveillance” package; 9999 permutations).
[b] Data were log-transformed to achieve normality.
Organic (C): Organic certified farms
Organic (T): Organic transitional farms

Table S4. Bat activity (Mean ± SE) in 22 pair sites of organic and conventional citrus orchards (11 pair sites organic-certified vs conventional; 11 pair sites organic-transitional vs conventional).

Taxa	Conventional Citrus Orchard	Transitional Organic	Certified Organic	Total	Total (raw data)
Eptesicus-Nyctalus spp.	0.55 ± 0.8	0.18 ± 0.4	1.27 ± 2.45	0.64 ± 1.38	28
Tadarida teniotis	0 ± 0	0.18 ± 0.6	0.09 ± 0.3	0.07 ± 0.33	3
Pipistrellus pipistrellus	27.82 ± 39.81	27.73 ± 66.6	62.27 ± 131.59	36.41 ± 77.85	1602
Pipistrellus kuhlii	146.73 ± 157.71	68.27 ± 66.68	254.82 ± 270.97	154.14 ± 186.45	6782
Hypsugo savii	9.23 ± 13.28	2.36 ± 3.17	10.73 ± 15.15	7.89 ± 12.36	347
Pipistrellus pipistrellus-kuhlii	2.64 ± 2.13	2.91 ± 2.12	4.45 ± 3.8	3.16 ± 2.68	139
Pipistrellus kuhlii-Hypsugo savii	14.36 ± 19.44	5.64 ± 8.59	31.36 ± 40.48	16.43 ± 25.92	723
Miniopterus schreibersii	3.5 ± 5.47	2.45 ± 4.11	1.91 ± 3.45	2.84 ± 4.67	125
Myotis spp.	0.77 ± 1.31	0.36 ± 0.5	1.27 ± 2.15	0.8 ± 1.44	31
Plecotus spp.	0.64 ± 1.71	0.27 ± 0.65	1.64 ± 5.1	0.8 ± 2.8	35
Rhinolophus hipposideros.	0.14 ± 0.35	0 ± 0	0 ± 0	0.07 ± 0.25	1
Rhinolophus ferrumequinum					1
Rhinolophus blasii					1
Total bat activity	206.59 ± 210.58	110.73 ± 101.98	369.36 ± 402.55	223.32 ± 265.71	9826

Table S5. Mean (± SD) number of structural orchards features for each farming system (Conventional vs. Organic (T) vs. Organic (C)).

Structural orchard features	Conventional	Organic (T)	Organic (C)
% Ground vegetation cover (GVC)	27.64 (±22.92) NS	28.64 (± 26.38) NS	27.64 (± 28.81) NS
Tree Height (m)	3.71 (± 0.91) NS	3.68 (± 0.93) NS	3.86 (± 0.75) NS
Area (ha)	0.75 (± 0.63) NS	0.7 (± 0.35) NS	0.9 (± 1.06) NS

Organic (T): Organic -transitional farm; Organic (C): Organic-certified farm.

GVC: Amount in percentage of any ground free growing herbaceous vegetation

NS: not significant

Table S6. Description of the reclassified CORINE Land Cover data 2006 (CLC) used to (i) characterize the different habitats surrounding the citrus orchard plots and (ii) perform the landscape analysis.

CLC code	CLC description	New code	New description
112	Discontinuous urban fabric	1	Urban area
121	Industrial or commercial units	1	Urban areas
122	Road and rail networks and associated land	1	Urban areas
131	Mineral extraction sites	1	Urban areas
141	Green urban areas	1	Urban areas
142	Sport and leisure facilities	1	Urban areas
211	Non-irrigated arable land	2	Arable lands
212	Permanently irrigated land	2	Arable lands
221	Vineyards	3	Vineyards
222	Fruit trees and berry plantations	4	Fruit Orchards
223	Olive groves	5	Olive groves
241	Annual crops associated with permanent crops	6	Other agricultural areas
242	Complex cultivation patterns	6	Other agricultural areas
243	Land principally occupied by agriculture, with significant areas of natural vegetation	6	Other agricultural areas
311	Broad-leaved forest	7	Mixed and deciduous forests
312	Coniferous forest	8	Coniferous forests
313	Mixed forest	7	Mixed and deciduous forests
321	Natural grasslands	9	Semi-natural areas
322	Moors and heathland	9	Semi-natural areas
323	Sclerophyllous vegetation	9	Semi-natural areas
324	Transitional woodland-shrub	9	Semi-natural areas
333	Sparsely vegetated areas	9	Semi-natural areas
511	Water courses	10	Freshwater surface
512	Water bodies	10	Freshwater surface

Table S7. Results of the Mantel test performed to investigate spatial correlation (*r*) of the response variables. No correlation was found (empirical *P*-values > 0.05; 9999 permutations).

Model	Response variable	|*r*|	*p*
**Model 1: Organic-certified vs. Conventional**			
	P. kuhlii	0.102377	0.2094
	P. pipistrellus	-0.1447137	0.8968
	H. savii	-0.1628165	0.9720
	M.schreibersii	-0.1805727	0.9640
**Model 2: Organic- transitional vs. Conventional**			
	P. kuhlii	-0.058001	0.8338
	P. pipistrellus	-0.07382838	0.9203
	H. savii	-0.1243565	0.9045
	M.schreibersii	-0.01025743	0.4961
**Model 3: Organic- transitional v. Organic- certified + cofounding variables**			
	P. kuhlii	-0.01070954	0.5183
	P. pipistrellus	-0.04875028	0.6823
	H. savii	0.06428965	0.2423
	M.schreibersii	-0.03403262	0.6738

Table S8. Description of the candidate models used to assess bat responses to farming system (matched design: conventional vs. organic certified and conventional vs. organic-transitional) on its own or in interaction with the amount of semi-natural habitats within 2-km radius buffer scale (i.e. proxy of landscape complexity). The null model is also displayed for comparison.

Response variable	Model	AICc	ΔAICc	df	Weight
P. kuhli	***Organic certified vs Conventional***				
	Null model	271.6	5.9	3	0.04
	Model 1: Farming system	265.8	0.0	4	0.75
	Model 2: Farming system * landscape complexity	268.3	2.6	6	0.21
	Organic transitional vs. Conventional				
	Null model	261.2	1.6	3	0.30
	Model 1: Farming system	259.6	0.0	4	0.67
	Model 2: Farming system * landscape complexity	265.5	5.9	6	0.04
P. pipistrellus	***Organic certified vs Conventional***				
	Null model	199.8	0.0	3	0.61
	Model 1: Farming system	201.1	1.3	4	0.32
	Model 2: Farming system * landscape complexity	204.2	4.4	6	0.07
	Organic transitional vs. Conventional				
	Null model	479.1	290.7	2	<0.001
	Model 1: Farming system	188.5	0.0	4	0.95
	Model 2: Farming system * landscape complexity	194.2	5.7	6	0.06
H. savii	***Organic certified vs Conventional***				
	Null model	145.7	0.0	3	0.81

	Model 1: Farming system	148.6	2.4	4	0.19
	Model 2: Farming system * landscape complexity	156.7	11.0	6	0.00
	Organic transitional vs. Conventional				
	Null model	209.9	87.0	2	<0.001
	Model 1: Farming system	123	0.0	4	0.94
	Model 2: Farming system * landscape complexity	128.4	5.4	6	0.06
M. schreibersii	***Organic certified vs Conventional***				
	Null model	85.6	2.2	2	0.21
	Model 1: Farming system	83.4	0.0	3	0.63
	Model 2: Farming system * landscape complexity	86.1	2.8	5	0.16
	Organic transitional vs. Conventional				
	Null model	125.5	15.6	2	<0.001
	Model 1: Farming system	109.9	0.0	3	0.79
	Model 2: Farming system * landscape complexity	112.6	2.7	5	0.21

Table S9. Model selection from the global model that includes as explanatory variables farming system of organic-transitional vs. organic certified in interaction with the amount of semi-natural habitats as independent ones along with presence/absence of woody linear feature, ground vegetation cover, amount of semi-natural habitats, urban areas and distance to the nearest water bodies and Julian days. The most parsimonious models (*ΔAICc*<2), ranked by the second order information criterion *AICc* values, as well as the null model are shown. Presented are the number of parameters (K), the small-samples Akaike Information Criterion (AICc) and AICc weight (Wt). From the most parsimonious models, we only retained the models (indicated here in bold) with the lowest number of parameters and (whenever possible) that included farming systems (i.e. the variable of interest).

Response variable	Model	K	AICc	ΔAICc	Wt
P.kuhli	Null model	2	271.5	6.06	0.01
	Farming system + Ground vegetation cover	4	265.4	0	0.617
	**Farming system**	**3**	**266.3**	**0.95**	**0.383**
P.pipistrellus	Null model	2	199.8	11.65	0
	Ground vegetation cover	3	188.1	0	0.243
	Complexity at 2km + Ground vegetation cover	4	189.1	0.96	0.15
	Complexity at 2km + Julian day + Farming system	5	189.2	1.1	0.14
	Complexity at 2km + Julian day	4	189.4	1.32	0.125
	**Ground vegetation cover + Farming system**	**4**	**189.5**	**1.39**	**0.121**
	Ground vegetation cover + Linear features	4	189.7	1.57	0.111
	Ground vegetation cover + Linear features + Farming system	5	189.7	1.58	0.11
H. savii	Null model	2	128.3	3.78	0.013
	Linear features + Farming system	4	124.5	0	0.167
	Distance to water bodies + Linear features	5	124.7	0.15	0.155

	Distance to water bodies + Linear features +Farming system	4	125	0.46	0.133
	Linear features	3	125.6	1.05	0.099
	**Farming system**	**3**	**125.6**	**1.07**	**0.098**
	Ground vegetation cover + Linear features + Farming system	5	125.6	1.1	0.096
	Linear features + % Urban areas at 2km	4	125.8	1.25	0.09
	Linear features + % Urban areas at 2km + Farming system	5	125.9	1.34	0.086
	Complexity at 2km + Linear features + Farming system	5	126.1	1.57	0.076
M. schreibersii	Null model	2	87.3	1.88	0.054
	**Complexity at 2km**	**3**	**85.4**	**0**	**0.342**
	Linear features	3	85.7	0.31	0.293
	Complexity at 2km + Linear features	4	86.2	0.77	0.232
	Null model	2	87.3	1.88	0.134

Table S10. Results of the models retained after model selection (see table S9 for more details).

Response variable	Explanatory variable	Estimate	SE	Lower 95 CI	Upper 95 CI	*p*	R^2
P.kuhli	**Organic (T) vs. Organic (C)**	-1.3170	0.4251	-2.14595	-0.48806	**	0.41
P.pipistrellus	Organic (T) vs. Organic (C)	-0.7041	0.5300	-1.73760	0.32940		0.52
	**Ground vegetation cover**	1.2168	0.2464	0.73632	1.69728	***	
H. savii	**Organic (T) vs. Organic (C)**	-1.5126	0.5892	-2.66154	-0.36366	*	0.22
M. schreibersii	**Landscape complexity at 2km**	-0.8319	0.4236	-1.65792	-0.00588	*	0.19

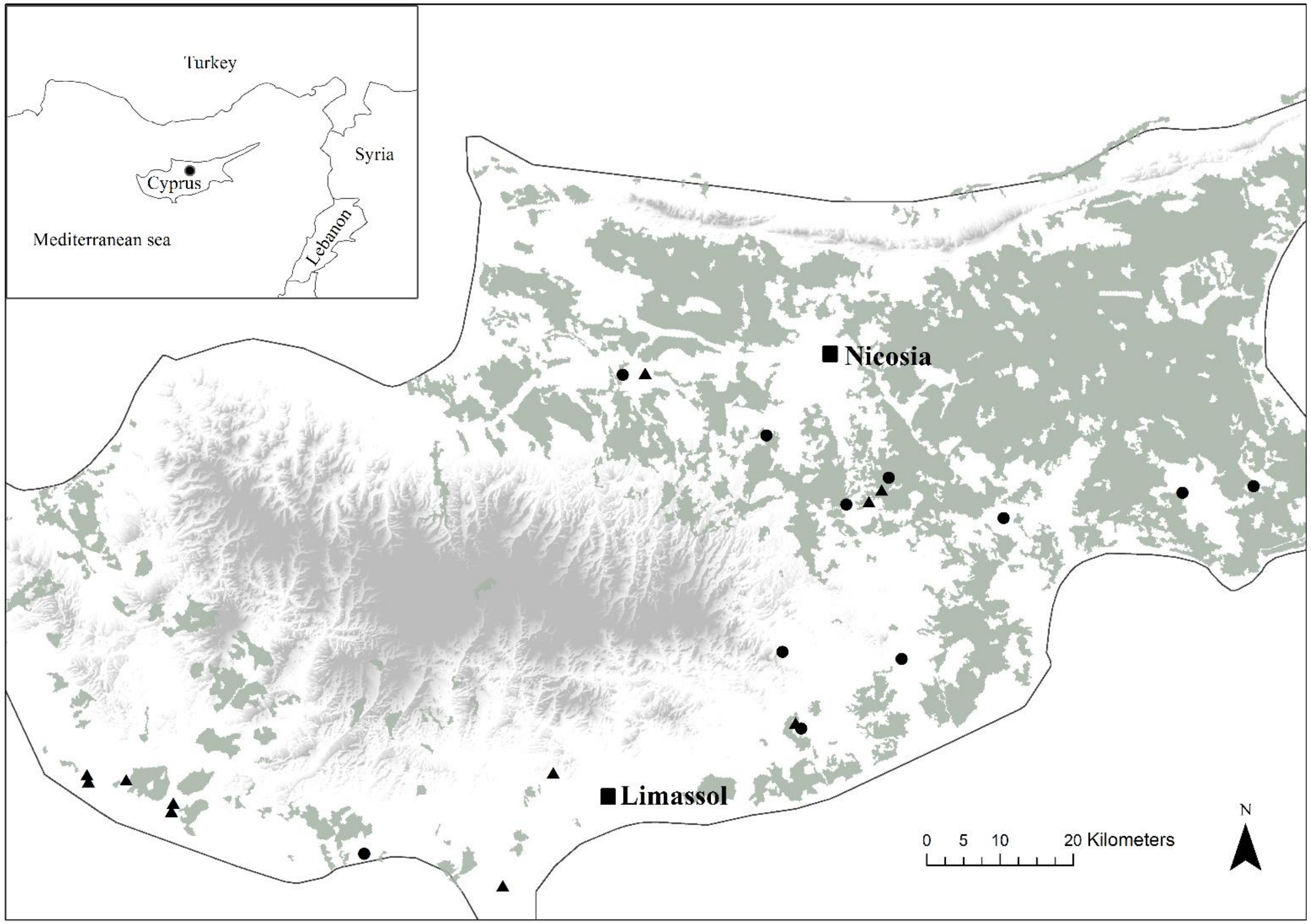

Fig. S1. Map of the study area This and areas covered surface of arable land. Acoustic sampling of bats took place in 22 paired sites in citrus orchards. Each symbol represents a pair. Pairs of conventional with organic certified farms are shown by circles, and conventional with organic-transitional farms by triangles. Arable land is shown in green and the elevation gradient in light grey, with darker grey corresponding to higher elevation.

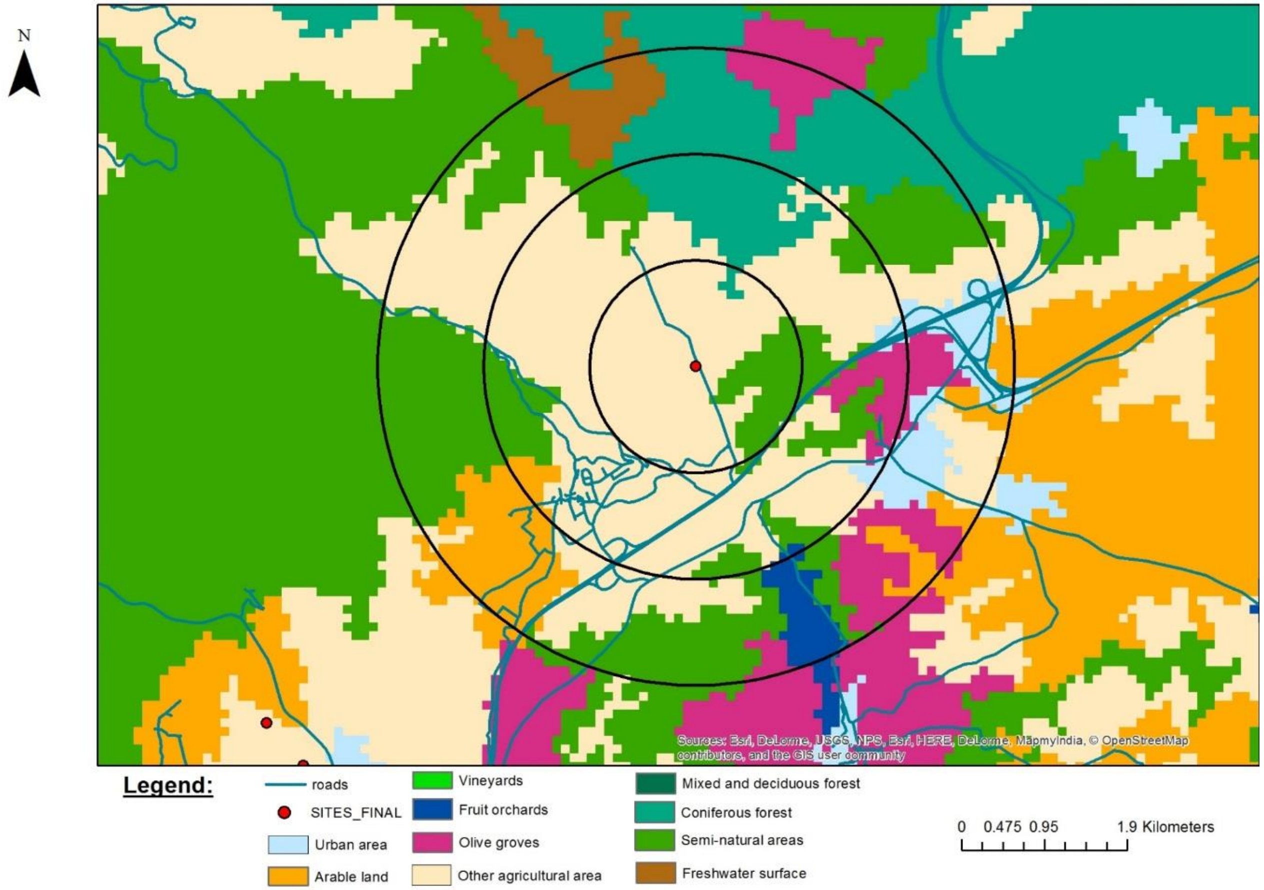

Fig. S2. Main land characterization based on CORINE Land Cover data 2006 (CLC) and example of one of 22 paired-sites and respective buffers of 1, 2 and 3 km used for landscape analysis.

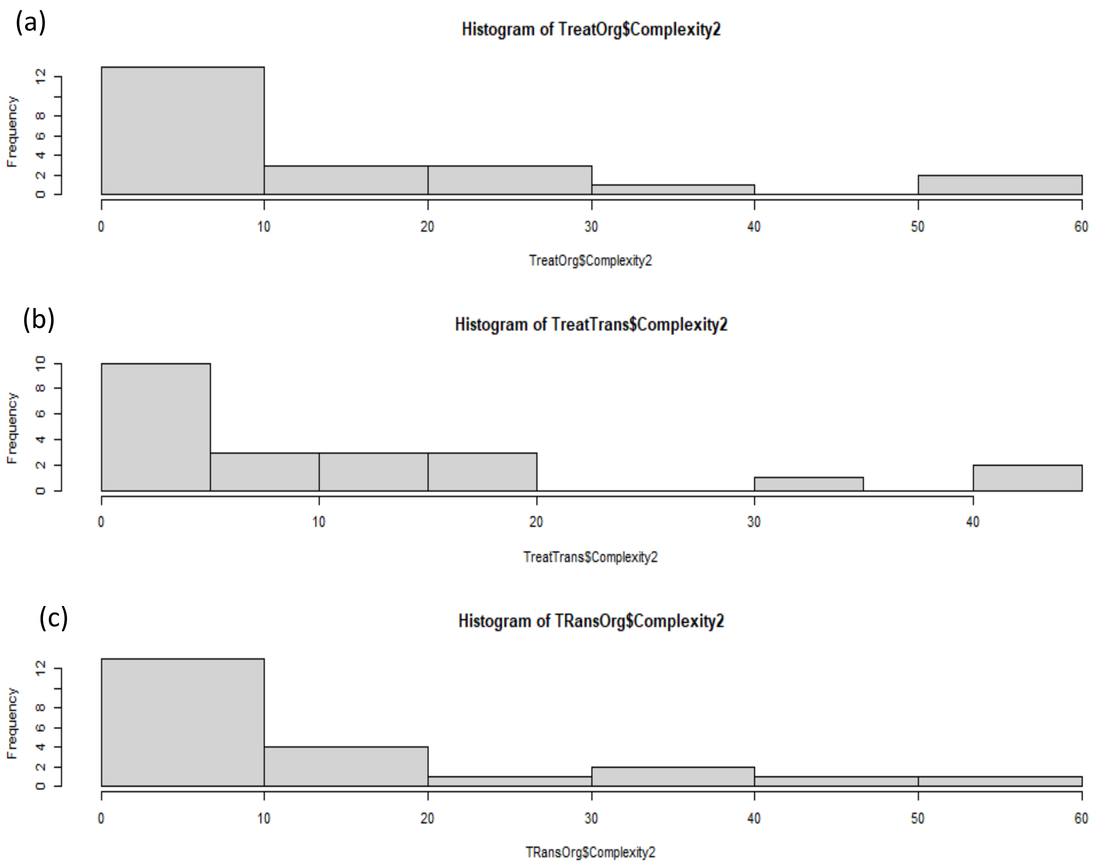

Fig. S3. Histograms showing the amount of semi-natural habitats (i.e..proxy of landscape complexity) at 2km radius buffer scale for each pair of farming system. (a) Organic-certified vs. conventional farming system; (b) organic- transitional vs. conventional and (c) organic-transitional vs. organic-certified farming system.
