# Supplementary material for: Transition to organic farming negatively affects bat activity
Source: J Appl Ecol. 2023 Jul 19;60(10):2167–76. doi: 10.1111/1365-2664.14468 (PMC10947233; doi:10.1111/1365-2664.14468)
Supplement: Supplementary file 2 — Appendix S2. Alternative statistical approach. [file JPE-60-2167-s001.pdf]

Appendix S2_Supplementary material

Transition to organic farming negatively affects bat activity

Penelope C. Fialas[1,2*], Jérémy S. P. Froidevaux[1,3,4], Gareth Jones[1,c] & Péter Batáry[5,c]

[*]Corresponding author: University of Bristol, School of Biological Sciences, Life Sciences Building, 24 Tyndall Avenue, BS8 1TQ Bristol, United Kingdom. Tel: +44(0)11.73.94.13.86. E-mail: pf16461@bristol.ac.uk

Bat echolocation call identification

Identification criteria were based on call characteristics (including social calls) provided by Russo and Jones (2002); Pfalzer and Kusch (2003); Obrist et al. (2004); Middleton and Nicolaou (2006). There are nine bat genera present in the study area (*Pipistrellus, Hypsugo, Myotis, Nyctalus, Eptesicus, Tadarida, Miniopterus, Rhinolophus and Plecotus*; and almost all can be identified based on characteristics of their echolocation calls. Due to the difficulties encountered in identifying echolocations from *Myotis* spp. (*Myotis capaccinii, Myotis nattereri*, *Myotis blythii, Myotis emarginatus*) and *Plecotus* spp. *(Plecotus austriacus, Plecostus kolombatovici)* to species level (Obrist et al. 2004), we identified these calls at the genus level. We were unable to differentiate between some calls belonging to *P. pipistrellus* and *P. kuhli* and therefore grouped them as *P. pipistrellus/P. kuhlii* (see Table 2), although it is likely that most individuals comprised *P. kuhlii*. The same approach was used for ambiguous calls of *Pipistrellus kuhlii* and *Hypsugo savii.* Similarly, it was not always possible to differentiate calls of *Eptesicus serotinus* from those of *Nyctalus leisleri and Nyctalus lasiopterus*, and we, therefore, grouped them as *Eptesicus-Nyctalus* spp.

Statistical analysis

Selection of covariates in the second set of analysis

To provide meaningful comparisons of effect size, all continuous variables in the second set of analysis were standardised (mean = 0 and SD = 1) prior to their inclusion in the models. To avoid multicollinearity and overfitting models, we conducted a series of GLMs using the response variables and the landscape metrics at each spatial scale as the only explanatory variables in each model. For each landscape-level metrics we selected the spatial scale with the largest effect size to be included in the final model. We then calculated the variance inflation factor (VIF) for each final model and variables that showed VIF > 3 were excluded for further analysis (Zuur et al., 2010).

Alternative approach

We used the whole dataset using pair type (conventional vs. organic-transitional, conventional vs. organic-certified) within period and **pair** within period were considered as random effects in the crossed-nested sampling design. We compared a series of candidate mixed models containing all possible preselected predictor variable combinations (see section above) using the *dredge* function (“MuMIn package; Bartoń, 2016) for model selection. We then adopted an information-theoretic approach based on AICc (Akaike Information Criterion corrected for small sample sizes) to select the most parsimonious models (Guthery et al., 2003) by using model averaging with the *model.avg* function in the MuMIn package.

Table S1. Standardized parameter estimates and associate standard error (SE) and 95% confidence intervals of the variables present in the most parsimonious models ($\Delta AICc < 2$) built to test the effects of farming system (conventional vs organic-transitional, conventional vs organic-certified), landscape characteristics, and citrus orchards structure on bat activity. Marginal (variance explained by the fixed effects only) and conditional (variance explained by both fixed and random effects) R^2 values are given. Explanatory variables displayed in bold represent significant variables for which 95% CI did not overlap zero. The spatial scale of each landscape attribute is given in brackets. Relative importance (RI) of a predictor represents the sum of *AICc* weights of all most parsimonious models containing that variable.

Response variable	Explanatory variable	Estimate (±SE)	Lower 95	Upper 95	RI
Total activity [1]	Organic (C) vs. Conventional	0.40 (±0.30)	-0.19	0.99	0.75
mR^2 = 0.26 | cR^2 = 0.55	**Organic (T) vs. Conventional**	-0.65 (±0.32)	-1.27	-0.02	0.75
	**Organic (T) vs. Organic (C)**	-1.04 (±0.39)	-1.82	-0.27	0.75
	**% Urban areas (3 km)**	0.39 (±0.17)	0.07	0.72	0.76
	**% Semi-natural areas (2 km)**	-0.41 (±0.20)	-0.80	-0.03	0.24
P. kuhlii activity [1]	Organic (C) vs. Conventional	0.48 (±0.32)	-0.14	1.11	1.00
mR^2 = 0.23 | cR^2 = 0.57	**Organic (T) vs. Conventional**	-0.72 (±0.34)	-1.38	-0.06	1.00

	**Organic (T) vs. Organic (C)**	-1.21 (±0.43)	-2.05	-0.36	1.00
	% Urban areas (3km)	0.33 (±0.18)	-0.02	0.69	0.38
	% Semi-natural areas (2 km)	-0.39 (±0.23)	-0.83	0.06	0.29
P. pipistrellus activity [1,a]	**% Urban areas (3 km)**	0.66 (±0.24)	0.19	1.12	1.00
mR^2 = 0.28 | cR^2 = 0.30	% Ground vegetation cover	0.35 (±0.34)	-0.31	1.01	0.29
P. pipistrellus activity [1,b]	Organic (C) vs. Conventional	-0.31 (±0.45)	-1.20	0.56	0.32
mR^2 = 0.20 | cR^2 = 0.36	**Organic (T) vs. Conventional**	-1.00 (±0.46)	-1.91	-0.09	0.32
	Organic (T) vs. Organic (C)	-0.69 (±0.59)	-1.85	0.47	0.32
	**% Urban areas (3 km)**	0.64 (±0.18)	0.29	1.00	1.00
H. savii [1]	**% Semi-natural areas (2 km)**	0.46 (±0.21)	0.05	0.86	0.72
mR^2 = 0.27 | cR^2 = 0.27	**% Ground vegetation cover**	0.45 (±0.21)	0.03	0.87	0.52
	P/A linear features	0.95 (±0.50)	-0.02	1.93	0.34
	**% Urban areas (2 km)**	0.43 (±0.19)	0.06	0.81	0.57
	Distance to water	-0.33 (±0.22)	-0.77	0.11	0.24

M. schreibersii activity [2]	% Semi-natural areas (2 km)	-0.65 (±0.34)	-1.31	0.01	0.62
mR^2 = 0.29 | cR^2 = 0.84	Organic (C) vs. Conventional	0.64 (±0.38)	-0.11	1.39	1.00
	**Organic (T) vs. Conventional**	-0.93 (±0.23)	-1.39	-0.47	1.00
	**Organic (T) vs. Organic (C)**	-1.57 (±0.45)	-2.45	-0.70	1.00
	P/A linear features	-1.08 (±0.58)	-2.21	0.05	0.44

Organic (T): Organic-transitional

Organic (C): Organic-certified

[1] GLMMs with a negative binomial distribution

[2] GLMMs with a Poisson distribution

[a] including one potential influential outlier; [b] excluding one potential influential outlier.

Table S2. Results of the information theoretical or "IT" model averaging approach across a candidate set of models of GLMMs built to assess the relationships between bat activity/species richness and farming system (conventional vs organic-transitional/organic-certified), landscape features and habitat structure. Only the most parsimonious models are shown (*ΔAICc*<2), ranked by the second order information criterion *AICc* values. Presented are the number of parameters (K), the small-samples Akaike Information Criterion (AICc) and AICc weight (Wt).

**Response variable**	**Model**	**K**	***AICc***	**Δ*AICc***	***AICc* Wt**
Total activity	Farming system + % Urban areas (3km)	9	567.23	0.00	0.52
	% Urban areas (3km)	7	568.70	1.47	0.25
	Farming system + % Semi-natural areas (2 km)	9	568.79	1.56	0.24
P. kuhlii	Farming system + % Urban areas (3km)	9	536.17	0.00	0.38
	Farming system	8	536.48	0.31	0.33
	Farming system + % Semi-natural areas (2 km)	9	536.71	0.54	0.29
P. pipistrellus[1]	% Urban areas (3km)	7	380.88	0.00	0.71
	% Urban areas (3km) + % Ground vegetation cover	8	382.68	1.79	0.29
P. pipistrellus[2]	% Urban areas (3km)	7	358.26	0.00	0.68

	% Urban areas (3km) + Farming system	9	359.73	1.47	0.32
H. savii	% Semi-natural habitats (2 km) + % Ground vegetation cover + P/A linear features + % Urban areas (2km)	10	275.03	0.00	0.18
	% Semi-natural habitats (2 km) + % Ground vegetation cover + % Urban areas (2km)	9	275.05	0.02	0.18
	Null model	6	275.74	0.71	0.13
	% Semi-natural habitats (2 km) + % Urban areas (2km)	8	275.79	0.75	0.12
	% Semi-natural habitats (2 km) + Dist. to water + % Ground vegetation cover + P/A linear features + % Urban areas (2km)	11	276.52	1.49	0.09
	Dist. to water	7	276.61	1.58	0.08
	% Semi-natural habitats (2 km) + % Ground vegetation cover	8	276.73	1.70	0.08
	% Semi-natural habitats (2 km)	7	276.80	1.77	0.07
	Dist. to water + P/A linear features	8	276.89	1.86	0.07
M. schreibersii	Farming system + % Semin-natural habitats (2 km)	8	201.44	0.00	0.39
	Farming system + % Semin-natural habitats (2 km) + P/A linear features	9	202.44	1.00	0.23
	Farming system + P/A linear features	8	202.74	1.30	0.20
	Farming system	7	203.01	1.57	0.18

[1] including one potential influential outlier; [2] excluding one potential influential outlier

References

Middleton, N. E., and H. Nicolaou. 2006. December 2006.
Obrist, M. K., R. Boesch, and P. F. Fluckiger. 2004. Variability in echolocation call design of 26 Swiss bat species: consequences, limits and options for automated field identification with a synergetic pattern recognition approach. Mammalia 68(4):307-322. doi: 10.1515/mamm.2004.030

Pfalzer, G., and J. Kusch. 2003. Structure and variability of bat social calls: implications for specificity and individual recognition. Journal of Zoology 261(1):21-33.
Russo, D., and G. Jones. 2002. Identification of twenty-two bat species (Mammalia: Chiroptera) from Italy by analysis of time-expanded recordings of echolocation calls. Journal of Zoology 258(1):91-103.
